# Inferential Integrity and Attention

**DOI:** 10.3389/fpsyg.2019.02580

**Published:** 2019-11-26

**Authors:** Carlos Montemayor

**Affiliations:** Department of Philosophy, San Francisco State University, San Francisco, CA, United States

**Keywords:** inference, rationality (descriptive and normative), consciousness, attention, agency theory

## Abstract

How should we define inferential reasoning in high-level cognition? Can non-conscious representations guide or even determine high-level cognition? If so, what are the properties of such non-conscious representations? Two contemporary debates on high-level cognition center on these issues. The first concerns the possibility of cognitive penetration, or the degree and extent to which high-level cognition influences or determines low-level cognition. The second focuses on the epistemic status of conscious cognition, and on whether or not non-conscious cognition could play a similar, albeit not as fundamental, justificatory role as conscious cognition. This latter issue is at the heart of the question concerning the epistemic status of conscious awareness. This paper focuses on the epistemic standard required for inference, or inferential reasoning, to count as justificatory. The debates on the epistemic status of consciousness and cognitive penetration typically assume such a standard because high-level cognition is associated with rationality, inferentially structured thought, and the epistemic responsibility one has for the conclusions drawn through one’s inferences. The paper proposes an account of *inferential-attention* that explains how cognitive penetration of non-phenomenally conscious cognition and perception is possible, and why there are unconscious processes that should be considered as essential components of high-level cognition. Sections “Defining Inference” and “Accuracy Constraints: The Agency-First Account of Inference” provide a theoretical framework for understanding the multiple criteria that an adequate account of inference and rational thought must satisfy. Sections “Attention: High- and Low-Level Inferential Cognition in Various Domains” and “Advantages Concerning Rule-Following and Rationality: Not Necessarily-Phenomenal Inferential Reasoning” articulate the inferential-attention account and explain how it meets the descriptive and normative criteria for epistemic responsibility and rationality. In particular, section “Attention: High- and Low-Level Inferential Cognition in Various Domains” defends an *agential* interpretation of inferential-attention, which offers a resolution of the tension between conservative or consciousness-based approaches to inference and liberal approaches that allow for types of unconscious or subdoxastic processes. An agency condition on inference explains how inference is a psychological process under the *control* of the agent, and at the same time, it satisfies the normative condition that an inference should be responsive to reasons or evidence by being cognitively available for personal level assessment and evaluation. The key is to identify this kind of epistemic agency with attention. Section “Advantages Concerning Rule-Following and Rationality: Not Necessarily-Phenomenal Inferential Reasoning” compares this inferential-attention account with an influential agential account of inference based on conscious intuition, and it argues that the former account is preferable. This section also demonstrates the significance of inferential-attention in higher cognition, even when it is non-phenomenally conscious.

## Introduction

Central questions about inferential reasoning concern its relation to conscious and unconscious cognition. Are there non-conscious representations driving (or determining) high-level cognition? If so, what are the properties of such non-conscious representations? At a neuroanatomical level, top-down influences from the prefrontal cortex regulate and contextualize sensorial filtering, inhibition, attentional selection, and task relevance, based on prior knowledge (Wimmer et al., 2015; Wells et al., 2016; Nakajima et al., 2019). The background knowledge involved in these cognitive influences includes emotional inputs (Song et al., 2017) and determines perceptual processing to a large extent (Lupyan, 2017). These executive control and decision-making influences on cognitive processing are an important characteristic of high-level cognition, but how exactly should we define them from a theoretical and epistemic point of view?

A classic approach to these questions is to define the interaction between prior knowledge and cognitive processing in terms of inferential relations (von Helmholtz, 1867/1910). It seems clear that a basic form of inference is involved in top-down influences, such as “if the task is *X*, then ignore feature *y* and select for feature *z*.” This approach, then, requires a precise definition of inference in order to understand the psychological influences of high-level cognition on lower level processing. There are two central debates about this issue in the contemporary literature. The first is the cognitive penetration debate, concerning the degree and extent to which high-level cognition influences or determines low-level cognition (Yeh and Chen, 1999; Macpherson, 2012; Zeimbekis and Raftopoulos, 2015). The second one concerns the epistemic status of conscious perception and cognition, and whether non-conscious cognition may also play a similar, albeit not as fundamental, role as explicitly rational and conscious thought. This latter issue is at the heart of the question concerning the epistemic role of consciousness. This paper focuses on the epistemic standard required for inference to count as justificatory (Siegel, 2017).

A common theme in these debates is the nature or type of inference required for high-level cognition. One strategy is to assume a restrictive notion of inference, according to which only conscious reasoning can give grounds for inferential reasoning that has an unquestionable epistemic status. A version of this view is that the type of reasoning from premises to conclusion that is unquestionably justificatory must be either explicitly endorsed by the thinker or somehow understood by the thinker as such—*as an inference*, the conclusion of which is accepted explicitly on the grounds that the premises are taken to be true. One option is that such explicit reasoning must be based on having cognitive access to the inference at the personal level, without necessitating phenomenal consciousness. An even more restrictive version holds that only phenomenally conscious reasoning can count as epistemically justified (see Boghossian, 2018, for a very useful categorization of inferential reasoning, including processes labeled as “inference” which according to Boghossian, should not count as inferences).

An alternative strategy is to assume a liberal notion of inference, according to which many instances of unconscious, automatic, and yet complex reasoning should count as inferential for epistemic purposes. The consequences of this view are epistemically revisionary, in the sense that many unconscious states that have the cognitive structure of an inference (i.e., they are selectively responsive to inquiries, based on premises or assumptions) count as reason-grounding and justificatory. This view is compatible with cognitive penetration, and it may also have the implication that cognitive penetration is common, perhaps even pervasive. It also entails the more surprising consequence that we are somehow epistemically *responsible* for inferential processes occurring outside the scope of our introspectively conscious, or intuition-based, cognitive control (see Siegel, 2017).

These rival conceptions of inference have fundamentally different consequences for the largely empirical issues regarding cognitive penetration and the epistemic nature of conscious perception and cognition (e.g., is there a natural boundary between perception and cognition; is inferential judgment fundamentally different from perceptual inference; are the different types of inference, at different levels of processing, radically different, or is there only a single type of *psychological process* that is genuinely inferential?). To address these questions productively, the stalemate between conservatism and liberalism about inference should be avoided. But it is hard to see how to avoid this stalemate if we appeal to the traditional approaches to epistemic justification, represented by the conservative and liberal views.

We have witnessed the clash of these rival approaches in several philosophical debates for at least four decades now and although there has been progress, it seems that internalist (conservative) and externalist (liberal) accounts may require new insights for the debate on inference and rational high-cognition to really move forward. This is, obviously, very important for psychology as well, since methodological questions concerning the design of experiments that investigate the nature of inference depend on the conceptual clarity with which experimental designs and results are interpreted. Avoiding a stalemate or a mere verbal dispute among the two dominant accounts of inference may thus require an alternative framework.

Is there a way to overcome these theoretical and methodological difficulties regarding the liberal and conservative approaches to inference? The present solution to this question offers a new way of satisfying the epistemic requirements for inference, based on *attention* as a form of *epistemic agency*. This proposal centers on the selective and guiding elements of attention. A moderate view on inference is defended, according to which unconscious processing may satisfy normative requirements for inference at the personal level, as long as agency is involved. An analysis of the conditions for inference that satisfy both automaticity and normative constraints is shown to justify an explanation of inference in terms of attention routines. This moderate perspective on inference incorporates the main normative requirements that the conservative view demands with respect to agency, but it avoids the problems that emerge from the introspective and phenomenal requirements of the conservative view.

Understanding the *psychology* of inference, especially the psychology of epistemically responsible inference, is a fundamental component of a satisfactory theory of rationality and high-level cognition. As Boghossian says: “in epistemology we are obsessed with the idea that there are better and worse ways for you to manage your beliefs; and that these ways reflect on your virtues as an epistemic agent.” (Boghossian, 2018, pp. 60–61). This is why delineating the boundaries of epistemically responsible inference (the type of psychological process that demarcates the realm of inferential reasoning and epistemic justification) is so important for any theory of high-level cognition.

From an experimental point of view, an account of inferential reasoning based on attentional integration at multiple levels of processing can explain, and be confirmed by, findings on common neural mechanisms for top-down control. As Song et al. (2017, 1) say, “numerous studies have recently suggested a shared neural circuitry underlying cognitive-emotional conflict resolution” (see Pessoa, 2008; Cromheeke and Mueller, 2014). This kind of conflict resolution, similar to the cognitive conflict resolution in the Stroop task, is attention dependent. In addition, attentional integrity has been confirmed as a crucial basis for uniform modulation and motor control (Lupyan, 2017; Rinne et al., 2018). Thus, the present proposal could help clarify how a unified neural mechanism for cognitive control can be understood theoretically in terms of the integrity of attention and inference.

## Defining Inference

What exactly should count as an inference and why is attention particularly useful to explain the psychology of inference? In order to avoid biasing the discussion toward the liberal or conservative view of inference, it is important to specify some conditions that the most basic kind of inferential reasoning must meet. Then one can explore how other conditions must be met for such reasoning to count as clearly epistemically justificatory, rather than merely “inference-like.” Although it is difficult to provide a neutral perspective on inference, most authors agree that an inference is a *psychological process* that provides an epistemically important outcome because of its cognitive structure (e.g., Siegel, 2017; Malmgren, 2018). There is also consensus about how this epistemic outcome must depend on a cognitively controlled *psychological action* that arrives at a conclusion in response to the content of the premises of the inference, which serve as reasons for the conclusion.

However, a topic of considerable disagreement is whether an inference requires access to the justificatory relations among propositional contents, such that a belief that *p* is justified for a subject only if it is based (at least partly) on the content of another proposition *q* for which the subject has justification. It seems that for the notion of inference to be explanatory useful, it needs to be a mental or psychological *action* under the control of the agent, rather than merely a *relation* among propositions (this is the difference between inference and argument—see Boghossian, 2018, for clarification). Propositional content and epistemically justificatory conditions must be in place in order to explain inferential reasoning. But on most accounts, an inference is a kind of mental process. I shall thus assume that a necessary condition on inference is that it is a psychological phenomenon that involves some kind of activity on the part of the epistemic agent.

Obviously, there is controversy surrounding the type of mental activity that best suits inferential reasoning; critically, can it be unconscious or is it necessarily phenomenally conscious? This issue is addressed in detail below, but for now, it is important to clarify that an inference is at the very least a psychological process guided by an epistemic agent in order to arrive at an epistemically important result. *A response to information is an inference only if it is the result of a guided psychological process. If the inference is adequate, then it provides an epistemic entitlement, typically justification*.

This is a rather minimal, necessary condition, on the justificatory status of inference, and a lot of the difficulties emerge from spelling out the details. But it is a good place to start. It allows us to now focus on two desiderata for the psychology of inference that directly bear on the debate between liberals and conservatives. The desiderata are that the definition of inference should be flexible in two ways. The first desideratum is that the definition of inference should allow for various psychological processes to count as inferential, and not just only consciously reflective belief. The second desideratum is that the definition of inference should allow for various styles of cognition.

Regarding the *various processes* constraint or desideratum, part of the justification for it is based on the fact that inferences are not merely relations among propositions. An inference is essentially a psychological process, and psychological processes can be quite diverse, so we need a criterion to isolate inferences from other processes that associate or relate propositional contents without being properly inferential. We do not want, however, this criterion to be too restrictive. On the one hand, we do not want subdoxastic, subpersonal, or strictly computational, associative, or merely representational processes to count as inferences. On the other hand, we do not want to restrict the notion of inference to a single type of psychological process associated with fully explicit and phenomenally conscious belief. For instance, mnemonic, emotional, implicit, heuristic, and perceptual processes may very well fall under category of “inference” and they should not be excluded from an account of the psychology of inference.

A plausible way of satisfying this desideratum is by defining inference as a psychological process under the control of an agent. This is still too liberal for conceptual and theoretical purposes because it includes mental states (such as jumping to conclusions) that should not count as inferences. But it is restrictive enough to prevent subdoxastic states from counting as inferential. It also allows for various inferential processes, as long as they are somehow under the control of the agent, and are responsive to contents in a way that leads to epistemic entitlements when the inference is adequate, precisely because the agent is in control. In some cases, an inference may even include the same content, but it might still establish an epistemically crucial relation between two different processes, for instance, from perception to belief (Siegel, 2017). An advantage of this kind of agential approach is that inferential processes at high and low levels of cognition may also include practical and ethical considerations, although I will not explore those issues here. This approach to inference, moreover, is fully compatible with very substantial types of cognitive penetration—high-level cognition may determine or guide low-level information processing, including perceptual processing, through the mental actions of an agent. The question is whether this notion of inference based on various psychological processes is too liberal. We shall soon return to this question.

With respect to the *styles of cognition* desideratum, the main idea is that human cognition should not be assumed to be the exclusive paradigm of inferential reasoning. This constraint seems initially much weaker than the *various processes* constraint. For how else should we explain inferential reasoning if not by reference to human psychology? But, as many authors have noted (Kornblith, 2012; Buckner, Forthcoming), research in animal cognition strongly favors an approach to inference that satisfies this constraint. Inference occurs in a robust, epistemically entitling manner, across various species with similar perceptual capacities to human perception. This should not be too surprising given that we share our evolutionary path with them. So, at the very least, given the abundant evidence of inferential reasoning in animals, an account of inference should allow for the possibility that there is some kind of inferential reasoning with a genuine epistemic upshot in non-human animals. These are truly epistemic achievements, in the sense that they satisfy an epistemic requirement that provides epistemic entitlements, such as an evidential or reliabilist standard.

Therefore, other things being equal, the desiderata above justify a liberal view of inference for the following reasons. Inferences are psychological processes that will, at the very least, resemble psychological processes in some species. This certainly tips the balance toward a liberal notion of inference when it comes to its *scope*. It still remains possible that other necessary conditions on inference will preclude the possibility of adopting a liberal notion, thereby reducing the scope of inferential reasoning to perhaps just humans. But these two desiderata are based on the necessary condition that inferences are psychological processes and, therefore, they have significant initial support because similar psychological process have been identified in many non-human species. Ideally, and in accordance with the *various processes* desideratum, inferences should not be restricted to a single class of psychological processes associated exclusively with explicitly endorsed belief, and our notion of inference should be compatible with the substantial experimental data emerging from psychology, demonstrating inferential capacities in non-human animals.

The *various processes* desideratum is theoretical; it is not based on psychological evidence. This desideratum centers on the concept of inference—the type of psychological process we shall define as inference. The *styles of cognition* desideratum is based on empirical considerations, but these considerations are particularly powerful, given the shared evolution of our cognitive capacities (see Haladjian and Montemayor, 2015, for why evolution gives support to an attention-based approach). In this sense, this desideratum is not simply a demand for empirical evidence because it receives support from considerations concerning a plausible theory of our psychology and its evolution. The following are additional considerations in favor of these desiderata. Overall, they favor a liberal view.

### Automaticity

If we adopt a liberal notion, then inferential processes can account for automatic and immediate forms of epistemic entitlement that would otherwise be categorized as non-inferential.

### Complexity

If we adopt a liberal notion, then we can account for high degrees of complexity in reasoning with respect to various types of mental processes, from basic perception to abstract thought, which should be categorized as inferentially integrated with one another.

### Integration

If we adopt a liberal notion, then we can account for various forms of epistemic influences, including cognitive penetration.

These are three aspects of a liberal view that are based on the desiderata above. Before presenting some fundamental challenges to the liberal view, it is important to emphasize that these desiderata were presented in order to flesh out one of the key elements of the necessary condition for inferential reasoning, namely, that inferences are psychological *processes*.

## Accuracy Constraints: The Agency-First Account of Inference

The previous section shows that if one considers inferences as psychological processes, then one should favor a liberal approach. The situation is quite different, however, with respect to the requirement that the outcome of such a psychological process, when adequate, must provide unambiguous and robust types of *epistemic justification*, which is also a crucial component of the necessary condition on inferences. Here we find a fundamentally different type of disagreement which, according to many authors, clearly favors the conservative view.

Arguably, the central point of contention here is whether the psychology of inference necessitates a specific kind of *phenomenology*, or a specific kind of cognitive access that depends on subjective (even reflective) awareness. If so, the conservative argues, this requirement eliminates the relevance of the psychological desiderata because what is most distinctive about an inference is its unique normative status in epistemology, which depends on the phenomenal character or subjective experience of drawing an inference. Inference is fundamental to make sense of our rational practices, based on the notions of epistemic credit and responsibility. Conscious awareness, according to the conservative, is fundamental to the type of access needed for drawing an inference. Thus, if it turns out that only human psychology can provide the *right kind* of conscious access that could ground inference, then we should ignore the empirical desiderata. Ultimately, if epistemic normativity conditions are not favored over psychological desiderata, then we lose track of the epistemic requirements that are the core of our concept of inference. To guarantee the epistemic standing of an inference, the subject must have conscious or reflective access to the process of how drawing the conclusion is based on the premises.

Given this line of argumentation, the conservatist confronts traditional difficulties concerning the nature of conscious epistemic access. Should internalism about justification be expressed simply as a supervenience condition requiring epistemic justification to depend exclusively on the internal properties of mental states regardless of their phenomenology or lack thereof, or should it also include mental types of a *specific kind*, namely states in which the subject has a unique and well supported access-relation to reasons or evidence? Should this state be one that is also reflective and always consciously available to the subject; should it also be accompanied by a very specific kind of phenomenology, for instance, the subjective experience of understanding?

I will not rehearse here the standard objections to positive responses regarding these questions, or list the difficulties that emerge from them. My goal is to classify these objections and responses in a way that helps move the debate forward. All forms of accessibilism about epistemic justification, strong or weak, justify their internalist requirements, against the psychological desiderata, by appealing to *epistemic norms*. In weaker versions, access to evidence is compatible with implicit, not necessarily conscious belief. In stronger versions, access needs to be grounded on the phenomenology of conscious reflection and the assessment of evidence or, in the case of inference, on the phenomenally conscious endorsement of the conclusion based on the premises. Thus in all versions, it is access to evidence, to reasons, to norms, or to all three that makes an agent *responsible* for her inferences. The agent deserves credit when the inference is drawn correctly and her inferences can be rationally evaluated because the agent is responsible for the inference. Although it is not trivial to define “access,” the fundamental assumption of all conservative versions is that cognitive access needs to be *personal level* access, as opposed to subdoxastic or merely representational psychological processing.

Let us, therefore, set aside for the moment the issue of whether or not access should be necessarily conscious (we shall return to this issue in section “Advantages Concerning Rule-Following and Rationality: Not Necessarily-Phenomenal Inferential Reasoning”), and settle now for the more common assumption among internalists that access must occur at the person level, and even be available for introspective reflection. By focusing on the less demanding versions of accessibilism we might find a more balanced perspective on the psychological and normative requirements for inferential reasoning. As is well known in the literature on rationality (e.g., Gigerenzer, 2008; Kahneman, 2011; Morton, 2012), the ideal account of inference would need to satisfy these two requirements:

**Descriptive adequacy**: the access requirements on inference must be actually true of human psychology.

**Normative adequacy**: the access requirements on inference must satisfy conditions for rationality, evidential support, and epistemic justification.

One way of satisfying *normative adequacy* is to postulate additional necessary conditions on inference, such that the evidential and justificatory support for a belief is antecedently grasped or assessed by the subject. This epistemic access to the propositions or beliefs that epistemically support a belief explains why the agent is justified in believing it—mere propositional support does not entail doxastic or endorsed-belief justification, but it provides rational grounds for the inference. This approach is in line with conservatism because it denies the status of “inference” to reasoning that is inferentially structured, but unavailable for scrutiny and person-level evaluation. Yet, it grants that inferentially integrated but inaccessible states *can* play a kind of justificatory role, only one that is *non-rational* or available to personal level scrutiny (Malmgren, 2018).

An alternative approach is to grant epistemic standing to such “non-evaluable” or non-accessible inferences, which brings us back to the issue of how flexible the account of inference must be. This second option allows for inferential reasoning that is not available at the personal level to count as epistemically evaluable, in positive and negative ways (Siegel, 2017). This approach is certainly more liberal. Both proposals aim at striking a balanced view that could satisfy descriptive and normative adequacy. But both have the limitation that they end up favoring a version of conservatism and liberalism. The impasse remains.

It seems that as long as the emphasis is on conditions for inference *alone*, as a *process* that needs to be understood in terms of either abstractly formulated necessary conditions for access and evaluation, or epistemically supportive mental events that should count as inferentially integrated based on the characteristics of an actual psychological process, the impasse will remain. The psychological conditions must be fleshed out and developed in a way that the normative conditions are also clearly satisfied. The descriptive and normative adequacy conditions seem to pull in opposite directions, but only if essential considerations about *epistemic agency* are ignored; or so I will now argue.

On *all* accounts of inference, although this is rarely noticed or expressed explicitly, agency is fundamental, at least in a “formal” way—as an implicit aspect or element of the definition of the process that should count as an inference. The problem is that agency is never considered as the *defining* feature of inference, and the focus is instead on access to information and conscious states. But most authors would agree that an inference is a psychological process that a subject is somehow *in control of*. Conscious control at a personal level (rather than a subdoxastic or subpersonal level) seems crucial for the conservative view. And some kind of personal level or agential control, even if it is unconscious, is also required for the liberal view because an inference is drawn by an *epistemic agent*, and not by a module or subdoxastic computational component of the agents’ architecture. Otherwise it seems implausible to attribute the inference to the agent. The present proposal is to explicate the psychological requirements of epistemic agency in inferential reasoning in order to address the descriptive and normative adequacy conditions at once. Call this the *agency-first approach* to inference. Unlike the “process” or “conditions for access” approaches, the agency-first approach restricts the relevant type of psychological processes that should count as inference only to those processes that count as genuine exercises of agency or mental actions of a subject.

An agency-first condition on inference explains how inference is a psychological process under the control of the agent, and at the same time it satisfies the normative condition that an inference should be responsive to reasons or evidence available for personal level assessment and evaluation, at least in principle (see section “Advantages Concerning Rule-Following and Rationality: Not Necessarily-Phenomenal Inferential Reasoning” for details). The key, therefore, is to identify a *psychological process that qualifies as epistemically agential control*. The novel aspect of the present proposal is to identify this kind of epistemic agency with *attention* (for the purposes of cognitive penetration, particularly the so-called top-down attention).

A condition based on agency is as follows: *A psychological process is an inference only if it is under the cognitive control of an epistemic agent*. If one adopts the *inferential-attention* view, this condition states that *a psychological process is an inference only if it is under the cognitive control of an epistemic agent, understood in terms of her attentional capacities.* This satisfies the *descriptive adequacy* condition by identifying a well-known psychological process that explains the kind of epistemic control needed for inferential reasoning. How about the normative adequacy condition? Once agency and cognitive control are considered as central, a performance normativity account of epistemic justification can satisfy the normative condition (for a comprehensive virtue-theoretic account of epistemic agency based on attention routines, see Fairweather and Montemayor, 2017).

Very rarely have virtue approaches been considered as relevant in the literature on the nature of inference. It has been generally assumed by defenders of the conservative view that only introspective conscious control can satisfy normative requirements because only introspective conscious access can guarantee the availability of contents for inferential reasoning and epistemic evaluation. But this is, at best, an incomplete picture of how inferential abilities satisfy the normative constraint because one still needs an account of how introspective abilities ground and integrate inferential abilities and mental processes. Introspection, if it is indeed an epistemic skill, is quite different and independent from inference (many authors are indeed skeptical about the epistemic status of introspection, with respect to both its nature and reliability; see for instance Dretske, 2003, 2012; Reginster, 2004; Kornblith, 2012; for an opposing account see Moran, 2001).

Phenomenal consciousness, or the subjective qualitative character that accompanies conscious experience, by itself, falls short of satisfying the normative condition because one needs to show how merely *experiencing* a conscious content guarantees *the cognitive ability to infer*. It is dubious that merely being in an experiential state with a particular phenomenal character will suffice to explain an ability, because abilities must be defined in terms of success conditions, not merely experiential ones. For instance, even if you have the experience that you should be able to hit a homerun, the contents of your experience do not necessarily entail any connection between your experience and the facts that must obtain for you to have the ability to hit homeruns. Your conscious experience that you can hit homeruns is relevant for baseball playing only if it is associated with the ability to hit homeruns.

Inferences are not just experiences or even thoughts with qualitative character. Rather, they are essentially abilities to draw conclusions from premises under the control of an agent: a kind of mental action. Thus, reflective conscious thought and introspection, by themselves, are *insufficient* to explain the kind of ability needed to infer. One needs to appeal to some kind of mental action or skill at the personal level to sufficiently explain inferential reasoning. Although there are considerable grounds for skepticism about conscious introspection as a condition for inference, the emphasis here is on the need to explain inference as an ability in the first place. The point is not that conscious awareness is *irrelevant* in inferential reasoning, but rather that it is *insufficient* to explain inferential abilities.

In spite of the fact that an agency-first approach to rational inference has not been the main focus of attention in the literature on inference, the central role of abilities features in some recent discussions about epistemic justification, particularly concerning how to confront skepticism (Lasonen-Aarnio, in press; Williamson, in press). For instance, Lasonen-Aarnio (2010) draws a useful distinction between rationality and reasonableness in terms of a success condition: rationality necessitates success (basing belief and inference on evidence) while reasonableness only requires a competence or disposition to do so. Our inferential abilities must *succeed* in typical conditions for them to satisfy the normative constraint and count as rational. These normal conditions must involve the control and guidance of the agent, but they *need not* involve conscious introspection, reflection, or awareness—although conscious introspection *can* play an important role. This means that the success required for rational inference depends fundamentally on the agent’s cognitive control through her abilities to succeed, and not necessarily on whether or not the agent is in a particular kind of conscious state with a specific kind of phenomenal character.

Epistemic agency, as personal level cognitive control, is *necessary* for epistemic credit and responsibility, and this is a core assumption of performance-normativity approaches in epistemology (Sosa, 2007, 2015; Greco, 2010; Greco and Turri, 2011; Miracchi, 2015; Fairweather and Montemayor, 2017). But independently of the advantages of an ability or agency approach, the literature on inference shows that cognitive control is necessary to account for inference, either because of the conscious grasp of the inference by the agent or because of the unconscious mental action involved in moving from premises to conclusion. An ability- or agency-based approach, therefore, offers the best way to explain this type of cognitive control. Reflective or conscious endorsement might be sufficient for epistemic normativity, if it is accompanied by ability and cognitive control, but it is not necessary because there are other types of epistemic agency, as explained below. An ability-based approach to inference can meet normative standards for knowledge and rationality without entailing problematic assumptions about conscious reflection or access (Fairweather and Montemayor, 2017).

One can go further, and argue that an ability-based approach is superior to current approaches to inference, partly because the success condition on rational belief is built into an agency account. While this might be the case, all that is needed for present purposes is to show that a virtue account of inferential abilities suffices to explain their epistemic status. This has the significant advantage that one can give an account of inference that is compatible with the abundant evidence on unconscious reasoning and cognition. According to this account, unconscious or tacit cognitive processing can not only guide high-level cognition, but contribute to its epistemic status. Abilities provide an explanation of epistemic justification for inference that avoids the demanding requirements of necessitating conscious reflection and awareness. This approach has the consequence of accounting for the normativity of inference without collapsing into the impasse between conservatives and liberals. But the descriptive adequacy constraint still needs to be addressed. Explaining how an ability, virtue-theoretic approach, can satisfy this constraint (section “Attention: High- and Low-Level Inferential Cognition in Various Domains”) while also complying with rational norms (section “Advantages Concerning Rule-Following and Rationality: Not Necessarily-Phenomenal Inferential Reasoning”) is the purpose of the remainder of this paper.

## Attention: High- and Low-Level Inferential Cognition in Various Domains

According to the condition stated above, a psychological response to information is an inference only if it is the result of a psychological process under the control of the agent. If the inference is adequate, then it provides an epistemic entitlement, typically justification. Inference is a psychological process, but it cannot be merely a process if we want this process to satisfy the normative requirement. This requirement demands some kind of *agential control*. Otherwise, defining inference in terms of the reliability of a cognitive *process* falls way short of the standard for normative standing and epistemic responsibility. The challenge, then, is how to define inference as a psychological process without falling prey to traditional objections concerning the lack of normative standing. As mentioned, the present proposal is to define the psychological process involved in inference in terms of the agential control provided by *attention*.

Most authors define attention as a psychological process of selection (see Jennings, Forthcoming, for a historical account of how attention has been defined in philosophy and psychology). But attention is not merely a process of selection either (Mole, 2011; Watzl, 2017). As some authors have argued, it is a form of *mental action*, or selection *for action*, or *for a subject*, guided by motivations and intentions, even though many of these motivations and intentions are generally implicit (Wu, 2011; Jennings, Forthcoming). For present purposes, there is no need to endorse a view of attention in which attention is, necessarily, a psychological process of selection *by a subject* (a metaphysically robust “self”). What is needed is that attention be a process of selection that always occurs at the personal level, which includes motivations and goals (Wu, 2011; see Koralus, 2014a,b, for an erotetic approach to attention that explains selection and inhibition as question-sensitive; see Fairweather and Montemayor, 2018, for an account of the inhibitory functions of attention in terms of virtuous sensitivity and insensitivity to information). This characterization provides enough degrees of agency to satisfy the normative constraint in the sense that the agent is responsible for her inferential-attention routines. But can this account of agency through the cognitive guidance and control of attention uniquely satisfy epistemic standards? This question is addressed in detail in section “Advantages Concerning Rule-Following and Rationality: Not Necessarily-Phenomenal Inferential Reasoning”. Here it suffices to say that attention provides a type of guidance and control that is particularly relevant for epistemology (Fairweather and Montemayor, 2017).

Crucially, attentional selection at the personal level is essential to explain agential responsibility in multiple psychological studies (e.g., moral, epistemic, and even legal responsibility; see Jennings, Forthcoming). This is especially relevant when top-down attention routines modulate early processing. This modulation and guidance can have negative epistemic, as well as moral, repercussions, which make the agent accountable or responsible for such repercussions. For instance, recent studies demonstrate an alarming combination of unreliable epistemic guidance and morally reprehensible bias. Attention guided by racial bias produces unreliable inferences about the identity of objects (e.g., a gun versus hand tools) with socially disastrous consequences (Payne, 2001; James et al., 2013). Research has shown that similar effects drive attention in a “shooter task” (Correll et al., 2002), as well as judgments of criminality concerning objects (Eberhardt et al., 2004), age (Goff et al., 2014) and judgments about capital punishment (Eberhard et al., 2006), all in a racially biased way. These are inferences that are bad in at least two ways because they are epistemically and morally inadequate. This kind of attentional guidance is pernicious, but it is under the implicit or unconscious control of the agent.

Other effects of implicit inferences based on top-down attentional biases are less troubling from a moral perspective, but they could be problematic from an epistemic perspective because they might make attention unreliable. For instance, color perception seems to be susceptible of cognitive penetration (Macpherson, 2012). If the probabilistic process that determines the color I am seeing is red is specified by implicit inferences that are reliable enough to produce true belief, then such guidance is beneficial. But if the color I am seeing is determined by inferential influences that are pervasive and unreliable (for instance, I am more likely to see red when something looks like a tomato), cognitive penetration would spell disaster with respect to at least color perception (see Montemayor and Haladjian, 2017, for a critical discussion about the notion of cognitive penetration in the context of the functions of attention). Typically, the selective functions of attention routines are *virtuously sensitive* to reliable information—they tend to be epistemically adequate, because they ignore irrelevant information and are immune to frequent error by preventing an overwhelming influence of bias (see Fairweather and Montemayor, 2017, for the notion of “virtuous sensitivity”).

Attentional guidance is also fundamental for extremely skilled types of high-level inferential-attention, but perhaps such guidance is best understood as implicit or automatic inference, rather than cognitively penetrated perception. Below I provide an example of how this occurs in mathematics, where implicit inferential-attention has positive epistemic effects by virtue of *integrating* various kinds of attention. This would be a specialized type of high-level cognition dependent on inferential-attention without necessarily altering what one perceives. This is true, however, of all levels of attentional control. For example, attentional integrity and high-level executive function are fundamental for low-level motor dexterity and strength (Rinne et al., 2018). This kind of attentional integrity can be understood as *agential integrity*, which unifies the motor-control level with the executive-function level.

There is much to say about high-level attention for executive function, and also about why it need not be phenomenally conscious (this is the topic of the next section). For now, what is important about this discussion is that inferential-attention includes types of top-down influences on perception which may have either a detrimental or a positive effect. Since attention is a kind of epistemic agency that depends on selection, guidance, and motivation, the agent is *responsible* for the outcomes of her attention routines. Siegel (2017) discusses in detail these issues in terms of her theory of inference. But as Irving (2019) has argued, these effects are best understood as problems concerning *norms of attention*, or what I am calling the *rationality of inferential-attention*. Thus, Siegel’s account is compatible with the present proposal if understood, as Irving proposes, as an account of the rationality (and irrationality) of attention.

Attention comes in many varieties (e.g., object-based, spatial, feature-based), but as mentioned, all of them are forms of selection that can be characterized as capacities for sensitivity to contents and relevant information, as well as virtuous (or “good”) *insensitivity* to irrelevant or misleading information. This provides an inferential structure for eliminating defeaters and distracting information, and it makes possible successful performance across multiple situations. It also makes possible a kind of suppression, based on top-down functions, that is essential for optimal high-level cognitive processing, including memory. For instance, top-down attentional modulation determines non-phenomenally conscious memory trace formation and it also suppresses sensorial input to allow for high-level phenomenally conscious memory content (Jacob et al., 2015). I submit that something exactly analogous happens with inference. If this is correct, there is non-phenomenally conscious rational selection, guided by top-down attentional modulation, and at a different level of guidance there is phenomenally conscious rational cognition, determined by inferential reasoning that selects and guides by virtuously suppressing contents in order to allow for maximally specific (phenomenally conscious) access.

The integration of these attentional capacities at the personal level (rather than at the subpersonal level of cognitive processing not associated with the person’s guidance and control) constitutes a form of epistemic excellence that can be assessed in terms of good making features of the agent that explain her success in a variety of cognitive performances. Any attention routine starts with an input that triggers a guidance process of selection in order to obtain an answer. Success in epistemic tasks can thereby be attributed *to the agent* because of her capacities to integrate and attend to relevant features. Additionally, perceptual attention has assertive force because the contents one attends to are the basis for action and epistemic endorsement, as well as epistemic evaluation. These reliable attentional capacities are the basis for high-level rationality and cognition.

If inference is not conceived abstractly, merely as a psychological process with epistemic consequences, but rather as a specific kind of attention routine with a degree of assertive force associated with action and motor-control, then a moderate view on inference is possible, and for the reasons offered above, preferable. Epistemic endorsement and justification depend on the selective functions of attention. According to this account, the liberal is right in extending the scope of inference to its lowest bounds (see Siegel, 2017; Buckner, Forthcoming) and the conservative is right in demanding that inference be solely attributable to agents that have indisputable cognitive control over the inference, drawing the conclusion based on the premises of the inference (Boghossian, 2018). Attention is an ability that explains how inference is not merely a psychological process because it essentially involves a type of mental agency with a clear epistemic upshot: succeeding at solving an inquiry in an optimal and reliable manner with the guidance and control of the agent.

The definition of inference above states that a psychological process is an inference only if it provides an epistemic entitlement, typically justification. Why *typically*? This is because authors have divergent views about the nature of propositional content, the probabilistic or logical relations underlying inferential and conceptual reasoning, and also about the definition of “belief” required for inferential reasoning. Good inferences are sources of justification, but perhaps not all types of inferential reasoning are fully rational (Malmgren, 2018). The scope of the kind of inference discussed in this paper concerns *epistemically normative* justification. Perceptual and strictly inferential beliefs must meet a standard of justification to be epistemically adequate. If they typically or reliably provide epistemic entitlements, such as knowledge or justified belief, then they meet the epistemic norm. The present proposal is that perceptual and non-perceptual beliefs are justified through the reliable functions of a type of inferential-attention guided toward action and assertion, under the control of the agent, which explains why the agent deserves epistemic credit for having met the epistemic standard. The next section compares this account to an alternative account of inference. A full discussion of how inference operates in the moral, aesthetic, and practical domains will require extending the present account of inferential-attention to these normative domains—an issue that is beyond the scope of this paper.

It is important to consider, however, how exactly would inferential-attention operate in these other normative domains. This is an intricate topic. The output of inferential-attention in the epistemic domain is typically justified belief or knowledge because *truth* is the guiding norm: epistemic entitlement is explained in terms of inferential-attention guided toward truth and successful action. The output of inferential-attention in the moral domain is a belief or judgment concerning the goodness of a person or action, independently of truth or success. Morally justified belief is not at all merely true belief, just like actions are not good simply because they satisfy accuracy conditions. Conscious compliance with a moral norm, exclusively because of the norm, seems more fundamental in the case of morally guided attention than in the case of epistemic attention. But even this is controversial. Similar considerations are relevant with respect to the aesthetic domain, where attention seems to be more “free” from specific outputs than in the epistemic and moral domains and for this reason, it may be more independent from rational inferential reasoning, although it may not be independent from phenomenally conscious awareness.

But there is enough similarity in the structure of attention across domains to justify the hypothesis that inferential-attention will be capable of explaining rational inference in all domains. This similarity in structure can serve as the basis for a much more ambitious account of inferential-attention that encompasses the whole range of cognition based on unconscious attention and necessarily conscious attention. If attention is selection for action at the personal level, or selective information processing at the personal level, as argued above (see also Fairweather and Montemayor, 2017), then various forms of normatively relevant inferences can be explained through the functioning of attention. Such an account has potential for explaining various aspects of cognition in a theoretically robust way.

The main point is that high-level cognition for rationality and epistemically normative outcomes, according to the present account, need not be phenomenally conscious (there need not be any specific “what it is like” for the cognitive process to satisfactorily deliver such outcomes). High-level cognition in epistemology is compatible with an implicit, and phenomenally unconscious, kind of guidance that allows, nonetheless, for enough access and control over the inferential process. Although this view of inferential-attention does not necessitate phenomenal consciousness, it does not *exclude* it. In fact, this account of inferential-attention in epistemology is very well suited to explain the difference between optimal but unconscious reasoning and necessarily conscious reasoning which might be reliable only under very specific phenomenally specified conditions. Kahneman (2011), for example, emphasizes the unreliability of fast and frugal heuristic reasoning, while Gigerenzer (2008) defends such reasoning as optimal. Most authors agree that there is implicit or unconscious inferential reasoning, even if they disagree about the nature of phenomenally conscious rationality.

This proposal can, in principle, extend also to other species. The vast literature on inferential reasoning in animals will need to be explored in the light of the varieties of attentional-inferences that are possible in different domains (e.g., moral, practical, epistemic). The hypothesis that there are few uniquely human capacities, and the extent to which rationality is uniquely human, can be explored in more detail by specifying inferential-attention capacities in the moral, aesthetic, and epistemic realms. Moreover, the structural similarity among different kinds of attention is useful to draw a general distinction between good and bad inferences across different normative domains. Bad inference is, in general, unreliable inference, but depending on the normative domain, the unreliability of an inference may mean substantially different things. In the epistemic domain, reliable inference is truth-conducive (for instance, in the case of deductive inference, if the inference is drawn from a valid argument form, the inference is truth preserving—if the premises are true, the conclusion must be true). This kind of inference differs from the type of automatic inference in perception, but (1) both involve a rule concerning truth-conduciveness and (2) both require cognitive control, one explicit and the other implicit. Inferential reasoning in the moral domain, as mentioned, seems less capable of automaticity and it is also independent from truth-conduciveness.

Cognition, including the most paradigmatic examples of high-level cognition, is a kind of *attention-based dexterity* according to the present account. One can characterize “intellectually *responsible* intuitions” as a kind of high-level attentional dexterity. Think of Descartes’ discovery that one can prove truths about algebra through geometry and truths about geometry through algebra. There is no immediate relation, based on conscious reflection alone, that could justify investigating such proofs because the conscious access we have to visual and perceptual figures and forms in geometry seems entirely independent from the relation among ordered abstract entities, such as numbers. However, Descartes could “sense” that there had to be such a relation, based on his tacit knowledge of mathematics. Conscious awareness becomes guided by more tacit routines that help guide inquiry and arrive at a conclusion. There is a complex integration of inferential-attention routines involved in such an exercise. They include conscious representations with very specific phenomenology, like imagery, perception, and imagination. These contents are guided through implicit inferences that need not have any phenomenology (like the access we have to the relations among numbers). The cognitive agent, in this case Descartes, need not have any phenomenally conscious access to inferences concerning mathematical rules about algebra and geometry.

Groundbreaking mathematical discoveries are an exceptional case that illustrates how inferential-attention guides multiple forms of attention routines simultaneously. Take for instance what happens when one understands the Euclidean axiom of the parallels by “looking” at the parallel lines and “imagining” that they go all the way “up and down to infinity.” I bracket these terms (looking, imagining and projecting them to infinity) because none of these attention processes are *strictly* perceptual. They are all implicitly guided by a *rule* that defines the space of perceptual visualization (an infinite space!) as having zero curvature. One need not be consciously aware of this specific rule concerning zero curvature in order to follow it implicitly. Attentional guidance through perception and imagery thus allows us to understand the axiom of the parallels. This is not really a typical case of cognitive penetration (e.g., an implicit rule, emotion, or bias affecting how things *appear* to us in perception) because space never appears as infinite to us. But we clearly use this kind of top-down rational influence through inferential-attention to learn abstract knowledge, from basic mathematical and logical proofs to scientific theories. These are reliable inferences that prove that something is true (e.g., the axiom of the parallels is true in a space that has zero curvature) and they are guided by an abstractly but implicitly guided kind of attention to such figures.

In other domains, reliable abstract inference will not be essentially *truth-conducive*. The relevant notion of “reliability,” therefore, would need to be unpacked in terms of good-making features concerning moral, aesthetic, or prudential value. Although this is a complex task, attention and inference are flexible enough to explain these good-making features. In the moral case, one can easily conceive of a view in which inferential-attention is guiding our emotional and perceptual contents toward the needs of others, in a way that we become virtuously sensitive (morally virtuous in our selectivity), implicitly or explicitly, to the moral demands on our actions and the needs of others. A central question for an attention-based account is how to explain the centrality of norms and the grip of these norms on agents in at least the moral domain (and presumably the aesthetic domain). It seems that motivations, implicit and conscious, will need to play a key role in the explanation of normative guidance.

It is possible that normative guidance in some of these domains necessitates explicit conscious inference or phenomenally conscious attention (see Montemayor and Haladjian, 2015, for discussion). This would have substantial implications for research concerning the presence of moral and aesthetic capacities in other species, which presumably have phenomenal consciousness, but lack the capacity for linguistically guided inferences about explicit rational norms. There certainly are plenty of issues to investigate about how inference and attention operate across normative domains and across species. These are important topics for future research.

The next section examines an influential view of inference that emphasizes the role of phenomenal consciousness, and it argues that the inferential-attention account provides a more satisfactory explanation of the normative requirements for inference assumed by this prominent view, without necessitating phenomenal consciousness.

## Advantages Concerning Rule-Following and Rationality: Not Necessarily-Phenomenal Inferential Reasoning

Thus far, I have argued that an inferential-attention account of cognition satisfies several theoretical desiderata concerning the nature of inference, which are otherwise difficult to meet. The literature on inference emphasizes either the conscious and explicit rational endorsement of an inference or the flexible and automatic character of inferential reasoning. The first group of these views is associated with the highest forms of cognition and satisfies an epistemic norm for credit and responsibility while the second is associated with early cognitive processing that satisfies the criterion of empirical adequacy. I argued above that attention provides a new perspective that satisfies both constraints, the normative and the empirical. Crucially, the inferential-attention account provides insights into the nature of epistemic agency and it could in principle help explain inferential reasoning in other normative domains.

I shall now focus on an advantage of this inferential-attention account that concerns the notorious *problem of regress* about rule-following. The problem is that inference requires personal level cognition that seems to essentially depend on the *acceptance* of rules. Typically, this personal level acceptance is understood in terms of some form of decision or mental activity that *itself* is constituted by following a rule. This is what triggers the regress. More precisely, the acceptance of an inference depends on the intention or decision to draw a conclusion based on the premises of an inference, which is a kind of rational rule that must be correctly applied. It is this acceptance that explains why we draw the inference, and it also provides a reason for our doing so. But if an inference is already a rule of reasoning and there are rules concerning our decision to apply it to a concrete case, then there is the thorny question of our acceptance of the rules we employ in applying inferences to specific episodes of reasoning. Our acceptance of these rules, and the further rules that justify *their* application in particular cases, generates a regress because there seems to be no end to the process of determining which is the foundational rule that justifies all others.

Several difficulties emerge from this regress problem. I shall focus on problems associated with the type of mental act involved in the acceptance of a rule. Boghossian (2014, 2016, 2018) has defended one of the most detailed and comprehensive views about inference. He proposes that inference is a kind of *mental action*: “Inference, as I have characterized it, is mental behavior and, so, for it to make sense to hold you responsible for your inferences, inferring has to be something you *do*, and not just something that happens to you. It has to be a mental *action* of yours, something you have control over, and which you could have done differently, had you thought it desirable to do so.” (Boghossian, 2018, p. 60).

The fact that an inference is a mental action distinguishes it from an argument, because an inference is not merely a set of propositions, but fundamentally, a *movement* of thought from premises to conclusion (Boghossian, 2018, p. 55). But what is, exactly, this mental action? It must be an *intentional* mental action, precisely because you are responsible for it. But as Boghossian argues, these mental actions cannot be based on conscious or explicit intentions because tacit or implicit inference plays a central role in our epistemic lives (Boghossian, 2018, pp. 66–67). First, they need to be a mental or psychological type of process—an intentional mental action. Second, and more importantly, they need not be explicitly intentional—the intention for the mental action should not be exclusively defined in terms of the explicitly conscious intention to follow an inferential rule.

Boghossian argues for three features that demarcate the epistemically crucial psychological phenomenon we call “inference.” *Basing* determines that agents establish premises as the reason for believing the conclusion—the premises serve as the basis on which agents believe the conclusion. The *Quality* of an inference, given *Basing*, is that the belief drawn from the premises “can be assessed as resting on good or bad reasons.” *Responsibility* is based on these two properties, and determines your responsibility for reasoning well or badly—the assessment based on *Basing* and *Quality* determines an assessment of *your rationality* (Boghossian, 2018, p. 59).

According to Boghossian, these three features of inference apply to all and only those psychological processes that qualify as inference: fully explicit reasoning; inference without knowledge of the principle that allows for the transition from premises to conclusion; quick, effortless inference; and inference in children. Boghossian claims that so-called “inferences” that are subdoxastic or not at the personal level (like those involved in visual processing); inferential-like reasoning in *all* non-human animals; and artificially intelligent systems or computers, do not satisfy these three features, and therefore, should not fall under the epistemically fundamental category of *inference* (the one we associate with *responsible mental action*).

I certainly endorse Boghossian’s categorization of inferences as responsible mental actions, but the inferential-attention account could, contrary to what Boghossian believes, be extended to at least some animals because they are *agents*, as I explain below. However, this is not the key difference between Boghossian’s account and the inferential-attention account. The main difference between the inferential-attention account and Boghossian’s is that the former does not depend on phenomenally conscious states. But the two accounts are partially compatible because the inferential-attention account does not exclude the relevance of phenomenal consciousness. In fact, an inferential-attention account complements Boghossian’s because it solves the problems he raises in an *empirically plausible* way. In any case, independently of the empirical plausibility of the inferential-attention account, an attention-based approach to inference is superior to an *intuition-based* one (such as the one favored by Boghossian) for the following reasons.

A central challenge about the nature of inference concerns the “distance” between premises and conclusion, as our thinking moves from the premises to the conclusion. Boghossian illustrates this problem as follows: although Fermat’s last theorem *follows* from the Peano axioms, one cannot simply *infer* one from the other. This problem is related to the difference between an argument and an inference, but it is more intricate because this notion of “distance” between premises and conclusion in relation to mental action must be defined somehow. Boghossian writes,

“It looks as though what’s also needed is that the conclusion not be at too far a distance from the premise. But what does that mean? The only good answer that I can think of is that the step from premise to conclusion be such that the thinker have some *appreciation* that the conclusion does indeed follow from the premises. Of course, unless this condition is to generate a super-task, it had better be that, for a wide range of basic inferences, this appreciation is non-inferential in character.” (Boghossian, 2018, p. 60).

Boghossian’s solution to this difficulty is that, since the thinker must take the premise to support the conclusion, this “taking” must be “backed by an intuition to the effect that the taking is true.” (Boghossian, 2018, p. 60). This intuition-based approach is used by Boghossian to solve a lot more than the distance problem. In fact, the notorious regress problem is also tackled by Boghossian’s intuition-based approach, which appeals to the kind of understanding and appreciation provided by intuitions. Boghossian distinguishes two types of regress, which he calls “ingress regress” and “egress regress.” Ingress concerns the way in which we rationally *get into* the taking state. If we get to this state *via* an inference, which seems necessary since the state has a general content we must grasp, then it seems impossible to get into this state while avoiding regress. Egress involves the *transition* from the taking state to the conclusion—if it is through inference then it seems impossible to do so without regress.

Both of these problems, Boghossian claims, can be solved by appealing to conscious intuition. With respect to ingress, the reason why a thinker takes her premises to support her conclusion is because she has “the vivid intellectual impression” that whenever the premises are true, the conclusion must also be true (Boghossian, 2018, p. 62). The nature and importance of intuitions is briefly described as follows: “Taking states can seem like beliefs; but it’s important that, although they are belief-like, they are distinct from beliefs […] Underived taking states, that is, taking states not derived from other taking states, can only be entered into via intuitions (and not by testimony or inference).” (Boghossian, 2018, p. 62).

With respect to *egress*, Boghossian says that “we know of many examples of intentional states with general, conditional contents rationally controlling behavior without the benefit of inference.” (Boghossian, 2018, p. 63) He provides an example of how a tennis player implicitly controls her behavior without needing to conduct person-level inferences. The proposal is that the transition from the taking state to the conclusion can be in control of the agent *without necessitating an explicit inference* to guarantee mental control.

How about inferences in which, unlike inferences in mathematics or critical thinking, the thinker lacks both an explicit aim and an explicit “taking” state (namely, the other types of inference classified by Boghossian, including inferences in children?) Here Boghossian proposes that the three basic features of *Basing, Quality*, and *Responsibility* need to be understood in terms of goal-directed actions under the *rational control* of the thinker (Boghossian, 2018, p. 63). Something akin to conscious taking is needed to guarantee rational control. Boghossian argues that the solution is to propose taking states that are present *tacitly* (or implicitly). By relying on this tacit rational control, thinkers are not merely associating contents, but relating them under the tacit rational guidance that allows for transitions in thought of the form, “so” or “therefore.” Thus, quick and automatic inference can be under the intentional and rational guidance of the agent, even in the absence of a consciously grasped transition-rule or a conscious “taking” state.

This account of inference relies entirely on intuitions to solve the ingress and appreciation problems. It also depends partly on intuitions to solve the egress problem. Since the intellectual vividness of intuitions is what grounds the taking state in fully explicit inference, what is implicit must be precisely *this kind* of vivid intuitive support—the intuitive guidance is there; it just *becomes* automatic and habitual. These are insightful solutions to thorny and longstanding problems about the nature of inference and rationality. My focus now is on explaining how to complement and improve Boghossian’s comprehensive account of inference, by appealing to an attention-based, rather than an intuition-based, epistemology of inference.

Boghossian’s intuition-based approach certainly meets the conditions for stopping ingress and egress, and it also satisfies the normative constraints imposed by epistemic credit and rational evaluation. These are very substantial advantages of his approach. But is it *empirically plausible*? Can we *improve* on the empirical plausibility of this already powerful account of inference? Recall that empirical plausibility is an advantage of the agency approach, but perhaps this is an advantage only if epistemic agency is understood in terms of attention, instead of the more empirically controversial phenomenology of intuition, or vivid intellectual seemings. Let us first consider how attention would solve the problem of *appreciation*.

The personal appreciation of how the premises support the conclusion solves the problem concerning the distance between premises and conclusion. An intuitive-based account explains this in terms of the phenomenology (or what is it like) of the experience of taking the premise to support the conclusion. The most important aspect of the phenomenology of taking is that one has the vivid intellectual impression that whenever the premises are true, the conclusion must also be true—this explains the corresponding appreciation that the conclusion *follows* from the premises. The tacit guidance case lacks this explicit and conscious understanding, but it depends on it, as it has to be originally based on a conscious intuition that then becomes habitual. The question is whether this model of inference is capable of explaining tacit inferences in an empirically adequate way (i.e., should they all depend on intuition)?

One problem with the intuition-based account is that many inferences that we rely on to rationally guide our mental behavior are never based on intuition, and are tacit from the very beginning. Consider the inferences underlying our knowledge of syntax in language. It takes linguists years of training to explicitly appreciate the type of rules guiding syntactic inferences determining the grammaticalness or lack thereof of sentences. Typically, one only tacitly follows the principles guiding these inferences, without any conscious intuition concerning how one takes them to be the basis of conclusions about grammaticalness. So how is appreciation supposed to work for the young infant or the standard language speaker? This would be a kind of high-cognition inference driving grammaticalness which cannot be explained in terms of the vividness of an intellectual seeming or intuition. This type of inference is an *essentially implicit inference*—other examples of higher order cognition that rely on this type of inference include practical or inductive inferences or recognizing the speech acts of other people.

The inferential-attention account avoids this problem because high-level cognition is perfectly compatible with *non-phenomenally conscious forms of attention* that provide guidance and an implicit form of appreciation based on *attentional selection and salience*. This means that mental behavior can be rational even in the absence of “something it is like” to experience the vivid intellectual taking of the premises that support the conclusion. All that is needed is the guidance and control attention provides through selection and virtuous sensitivity. This can explain satisfactorily why essentially implicit inferences play a large role in our mental lives (see Wright, 2014; Siegel, 2017; Richard, 2019), and very likely, in the mental lives of non-human animals as well (Kornblith, 2012).

Thus, the inferential-attention account has the advantage over the intuition-based account of explaining essentially implicit inferences that require appreciation without phenomenal consciousness from the very beginning of mental development. Moreover, the inferential-attention account should be preferred from an empirical point of view because the psychology of attention is much better understood, and much less contested, than the psychology of intuitions or intellectual seemings. In fact, there is a whole branch of contemporary philosophy, namely experimental philosophy, that systematically criticizes the use of intuitions (and their phenomenology of certainty and truth) in philosophical analyses because intuitions can be shown to be unreliable in a variety of ways (see for instance Knobe and Nichols, 2008). Without endorsing an experimental-philosophical approach, I propose that the less controversial and well-verified psychology of attention should be the basis, instead of the psychology of intuition, of a satisfactory account of the psychology of inference.

But what about the non-trivial *normative* requirements that the intuition-based account clearly satisfies? Is the inferential-attention account capable of explaining the three key features of inference (i.e., *Basing, Quality,* and *Responsibility*)? I submit that the inferential-attention account can not only meet these three normative criteria, but that it can also provide an explanation of inferential mental action, in relation to these three epistemic criteria, that is superior to the intuition-based account. This does not mean that the attention-based account and the intuition-based account must necessarily be understood as rival accounts. In fact, if the requirement for conscious taking is circumscribed to only *explicit inference*, then the attention-based account can provide the ideal way to satisfy Boghossian’s normative constraints. In addition, the inferential-attention account can fully explain, and provide empirical support to, Boghossian’s *mental action* approach.

Inferential-attention can provide an explanation of the appreciation of how the premises support the conclusion by appealing to the selective and luck-eliminating functions of perceptual and cognitive attention routines. Attention selects information through virtuously sensitive information processes, and it ignores (or is virtuously insensitive) to irrelevant information, in a reliable and non-lucky way (Fairweather and Montemayor, 2017). This provides an explanation of appreciation that grounds justification for beliefs produced through inferential-attention. It also provides an explanation of the key properties of *Basing, Quality,* and *Responsibility*. The agent needs to take the premises to be the basis of her conclusion, determining that it provides a good reason to draw the conclusion, which she is responsible for drawing. As mentioned, this “taking,” according to Boghossian, must be “backed by an intuition to the effect that the taking is true.” But intuitions may lead one astray and essentially implicit inferences cannot be explained this way. The epistemic “force” or justification of an inference must find its source not just on the phenomenology of intuition but, fundamentally, on a *selective and luck-eliminating capacity* that leads to rational success.

Attention is perfectly suited to perform this role because the selective, luck-eliminating capacities of attention provide epistemic entitlements that intellectual seemings cannot explain by themselves. Attentional capacities are “luck-eliminating” because it is not by chance or accident that an attention routine one initiates through some implicit or explicit motivation satisfies the goal of moving our thoughts from premises to conclusion. This is, however, the only area of disagreement between Boghossian’s and the present proposal. This is why the inferential-attention account can be considered as an empirically adequate complement to, as well as a psychological explanation of, Boghossian’s mental-action proposal.

## Conclusion

More needs to be said about the intricate issues surrounding the relation between attention, inference, and rationality. But the outlines of a theory of rationality and the cognitive influence of high-level cognition presented above provide enough structure, I hope, to see the advantages of an inferential-attention approach to rationality. High-level cognition shapes many forms of early information processing and integrates low-level cognition through the guidance and selective functions of attention routines. Inferential-attention is a kind of dexterity and excellence of epistemic agents, who integrate their rational capacities at different levels of control and access.

There are key similarities between the inferential-attention account and prominent extant accounts. With respect to Siegel’s (2017) liberal inferential account of the rationality of perception, the present account endorses, and is fully compatible with, the agent-level guidance and responsibility that Siegel seeks in implicit reasoning beyond conscious awareness. The main difference is that for these accounts to be fully compatible, Siegel’s proposal must be interpreted as essentially dependent on the guidance and norms of attention, the way Irving (2019) suggests. Boghossian’s mental-action account of inference is also partially compatible with the present proposal. Here the central difference is that the inferential-attention account replaces the psychology of phenomenally conscious intuition with the more flexible, better understood, and well-confirmed psychology of attention.

Future studies on the psychology of inference should explore the normative force of different types of attention routines—or different norms of attention. One possibility is that some of these norms may enter into conflict with one another: an inference you might draw may be epistemically good because it is based on good evidence and produces more truth than falsehood, but it might be in conflict with how you should guide your attention toward the needs of others, in compliance with moral guidance. By contrast, it is possible that a comprehensive theory of the psychology of inference will require a comprehensive theory of the norms of attention, all of which should be compatible with one another and also be mutually supportive. These are crucial issues to investigate in future research.

## Author Contributions

The author confirms being the sole contributor of this work and has approved it for publication.

### Conflict of Interest

The author declares that the research was conducted in the absence of any commercial or financial relationships that could be construed as a potential conflict of interest.
